# Numerous Clones Resistant to *Phytophthora palmivora* in the “Guiana” Genetic Group of *Theobroma cacao* L

**DOI:** 10.1371/journal.pone.0040915

**Published:** 2012-07-26

**Authors:** Jean-Marc Thevenin, Vivien Rossi, Michel Ducamp, Fabien Doare, Virgile Condina, Philippe Lachenaud

**Affiliations:** 1 CIRAD, UPR Bioagresseurs : Analyse et Maîtrise du Risque, Kourou, Guyane, France; 2 CIRAD, UMR Ecofog, Kourou, Guyane, France; 3 CIRAD, UMR BGPI, Montpellier, France; United States Department of Agriculture, United States of America

## Abstract

Cocoa black pod rot, a disease caused by Stramenopiles of the genus *Phytophthora,* and particularly by the pan-tropical species *P. palmivora,* causes serious production losses worldwide. In order to reduce the impact of these pests and diseases, preference is given to genetic control using resistant varieties and, to that end, breeders seek sources of resistance in wild cocoa trees. For instance, surveys of spontaneous cocoa trees in French Guiana between 1985 and 1995 led to the collection of abundant plant material forming a particular genetic group (the “Guiana” group). Following numerous one-off studies demonstrating the merits of this group as a source of resistance to *Phytophthora*, this article presents the results of a comprehensive study assessing the resistance of 186 “Guiana” clones in relation to the Guianan strain (GY 27) of *P. palmivora.* This study, undertaken in French Guiana, using an efficient methodology (ten series of tests and a statistical test adapted to the ordinal nature of the data) confirmed that the “Guiana” genetic group does indeed constitute an important source of resistance to *P. palmivora*, though with some variations depending on the demes of origin. Numerous clones (59) proved to be as resistant as the SCAVINA 6 resistance control, whilst nine were statistically more resistant. The “Resistant” and “Moderately Resistant” Guianan clones totalled 108 (58% of the total tested). Some of the clones more resistant than SCAVINA 6 could be incorporated into numerous cocoa breeding programmes, particularly those that also display other notable qualities. The same applies for numerous other clones equivalent to SCAVINA 6, especially the “elite”’ clones GU 134-B, GU 139-A and GU 285-A.

## Introduction

Cocoa black pod rot, a disease caused by Stramenopiles of the genus *Phytophthora,* and particularly the pan-tropical species *palmivora*, causes serious production losses in all cocoa growing zones, varying from 30 to 90% depending on conditions [1]. Indeed, major losses result from infection of pods. Zoospores released in free water are spread to the pod. A brown lesion appears 2–3 days after penetration of the germ tube in the mesocarp and develops quickly into a large brown lesion. Beans can be more or less affected depending on the age of the pod when the attack occurs. Losses can also be indirect as *Phytophthora* can attack young leaves, flowers and roots, but also trunk and branches causing cankers.

Using varieties that are resistant to this scourge is an essential ecological and economic solution for integrated and sustainable control. Resistance of cacao to *Phytophthora* is of horizontal type. Only few resistant clones of various origins exist at the time being. Breeders are seeking sources of resistance in wild cocoa trees, in the species’ zones of origin (Amazonia and the Guianan Shield) in order to create, in their own producing countries, hybrids that will be locally tested. For instance, some wild cocoa trees in southeastern French Guiana, surveyed between 1985 and 1995 [2], [3], which make up the particular “Guiana” genetic group [4], have been subjected to early tests of resistance to *P. palmivora* in the laboratory [5].

The potential merits for genetic control of *P. palmivora* of some clones in the “Guiana” group, i.e. those originating from the Camopi and Tanpok river basins and named “GU” clones (“GU” stands for “GUyane”) [2], have already been revealed by early tests on leaf discs in the laboratory, in Ivory Coast, Ghana and France, but on limited samples [5]–[9]. In addition, tests carried out by CIRAD in Montpellier (France) with *P. megakarya*, a species existing only in Africa and currently in an invasive phase and tending to take over from *P. palmivora* on cocoa trees, involving 59 genotypes from 13 populations (originating from the basins of five rivers), revealed the exceptional merits of these cocoa trees, with 61% of the clones proving “Resistant” or “Very Resistant” [10].

Our aims were therefore (i) to carry out an exhaustive laboratory test on the “Guiana” clones held in the Sinnamary core collection (i.e. 186 clones) using a local strain of *P. palmivora* isolated in French Guiana (ii) to select resistant clones that could be used directly or as parents in breeding programmes.

## Materials and Methods

### Plant Material

The plant material studied consisted of 189 clones, comprising 186 clones of the “Guiana” genetic group and three representatives of other groups, used as controls.

The “Guiana” clones came from wild mother-trees collected in the basins of the Oyapok (called Kerindioutou in its upper reaches), Camopi, Euleupousing, Yaloupi and Tanpok rivers, in French Guiana [2], [3]. They were either cloned on site during the surveys or, mostly, selected and cloned in open-pollinated progenies after individual studies in the Sinnamary *ex situ* collection. The 186 clones represented 17 demes of natural populations, plus a subspontaneous clone (Table 1). These clones included 24 ortets,pre-selected after 10 years of observations at Sinnamary for their high productivity and their excellent field performance against pod rot diseases (Table 2).

**Table 1:** Distribution by deme of the 186 “Guiana” clones studied.

Deme	Nomenclature	Number	% of total
Borne 7	B7	7	3.8
Camopi 1	GU	27	14.5
Camopi 2	GU	1	0.5
Camopi 3	GU	16	8.6
Camopi 6	GU	1	0.5
Camopi 7	GU	19	10.2
Camopi 8	GU	2	1.1
Camopi 9	GU	40	21.5
Camopi 10	GU	1	0.5
Camopi 12	GU	5	2.7
Camopi 13	GU	10	5.4
Euleupousing	ELP	25	13.4
Kerindioutou	KER	19	10.2
Oyapok	OYA	3	1.6
Pina	PINA	1	0.5
Tanpok	GU	3	1.6
Yaloupi	YAL	5	2.7
Camopi 0	GU	1	0.5
Total		186	100.0

(Clone belonging to “amopi 0” is a subspontaneous individual of an undetermined deme but of local origin).

**Table 2:** List of resistant clones (ordered according to their effect in the ordinal probit model), their average score after 10 series of tests, and their deme.

Clone code	Clone name	Average	Deme	Resistance
35	GU 272-A	1.4	CAM 1	> SCA 6
97	ELP 8-A	1.38	ELP	> SCA 6
38	GU 278-A	1.48	CAM 1	> SCA 6
83	GU 326-A	1.53	CAM 9	> SCA 6
55	ELP 9-A	1.6	ELP	> SCA 6
45	GU 315-A	1.58	CAM 9	> SCA 6
51	GU 342-A	1.52	CAM 13	> SCA 6
138	GU 263-V	1.71	CAM 1	> SCA 6
10	GU 150-A	1.49	CAM 7	> SCA 6
37	GU 276-A	1.53	CAM 1	R
139	GU 265-V	1.68	CAM 1	R
66	GU 98-A	1.62	CAM 1	R
34	GU 270-A	1.63	CAM 1	R
102	GU 140-S	1.68	CAM 7	R
43	GU 295-A	1.69	CAM 13	R
8	**GU 143-A**	1.76	CAM 7	R
109	**GU 156-B**	1.74	CAM 1	R
168	ELP 15	1.7	ELP	R
67	GU 99-A	1.77	CAM 2	R
175	GU 174-A	1.77	CAM 9	R
59	ELP 20-A	1.81	ELP	R
15	GU 161-A	1.73	CAM 1	R
11	GU 152-A	1.79	CAM 7	R
60	ELP 22-A	1.86	ELP	R
7	**GU 139-A**	1.82	CAM 7	R
134	GU 255-V	1.78	CAM 1	R
58	ELP 18-A	1.86	ELP	R
26	GU 227-A	1.83	CAM 3	R
108	GU 155-A	1.94	CAM 8	R
107	GU 147-P	1.79	CAM 7	R
136	GU 257-E	1.8	CAM 1	R
24	**GU 216-A**	1.86	CAM 12	R
130	SCA 6	1.87		R
178	GU 306-A	1.88	CAM 9	R
137	GU 262-A	1.88	CAM 1	R
53	GU 347-A	1.89	CAM 3	R
91	SCA 6	1.88		R
106	GU 145-A	1.87	CAM 7	R
52	GU 344-A	1.92	CAM 13	R
6	**GU 134-B**	2.02	CAM 7	R
70	GU 102-A	1.92	CAM 1	R
18	GU 186-A	1.94	CAM 9	R
33	GU 266-A	1.93	CAM 1	R
87	ELP 11-A	1.99	ELP	R
122	GU 221-V	1.93	CAM 3	R
99	GU 123-V	1.96	TAN	R
88	ELP 14-B	1.99	ELP	R
72	KER 3	1.96	KER	R
56	ELP 10-A	2.01	ELP	R
5	**GU 134-A**	2.01	CAM 7	R
13	GU 156-A	2.03	CAM 1	R
86	ELP 1-A	2.02	ELP	R
160	KER 11-3-P	2.07	KER	R
116	GU 184-A	2.16	CAM 9	R
3	GU 129-A	2.06	CAM 7	R
41	**GU 285-A**	2.11	CAM 1	R
57	ELP 16-A	2.13	ELP	R
155	GU 334-A	2.06	CAM 13	R
148	**GU 303-B**	2.08	CAM 9	R
123	**GU 225-B**	2.2	CAM 3	R
189	KER 9	2.24	KER	R
49	GU 332-A	2.15	CAM 13	R
30	GU 240-A	2.26	CAM 3	R
142	**GU 285-C**	2.15	CAM 1	R
173	GU 160-A	2.14	CAM 1	R
132	GU 245-A	2.19	CAM 9	R
47	GU 325-A	2.16	CAM 9	R
146	**GU 297-B**	2.18	CAM 9	R
48	GU 331-A	2.17	CAM 13	R
62	ELP 35-A	2.17	ELP	R

The clones statistically more resistant than the SCA6 resistance control are scored “> SCA 6” and those equivalent to SCA 6 are scored “R”. The names of the clones in bold type are clones selected in the field for their high productivity and their low pod losses caused by rot diseases.

All necessary permits to conduct the surveys in the primary forest were obtained from the Representative of the French government, i.e. the Préfet de Guyane. No specific permit were then required for observations as the material was planted in the Cirad research station in French Guiana. *Theobroma cacao* is not an endangered or protected species.

The resistance control was the SCAVINA 6 clone ( =  SCA 6), the international reference used in tests involving *P. palmivora* [11]–[15]. This control was duplicated in the tests (2 samples). In addition, two other clones were used as controls: the resistant “Guiana” clone GU 255-V [8] and the moderately resistant Upper Amazon clone T60/887 [16].

Four clones from French Guiana were used as “susceptibility indicators”, to check that the inoculation tests were working properly: ELP 40-B and OYA 2-B, highly susceptible to *P. megakarya* [10], GU 138-A very susceptible to *P. palmivora* [9] and GF 24, classed susceptible to *P. palmivora* [8].

Clones were randomised in a plot maintained under artificial shade to homogenize the environmental conditions, particularly lighting [17]. It was a 0.135 ha budwood plot planted at Paracou-Combi in 2004–2005, with spacings of 2 m×1.5 m, where the trees were regularly pruned each year. Each clone was represented by two neighbouring trees, except SCA 6 and ELP 40-B represented each by two pairs of neighbouring trees. The edapho-climatic conditions at the Paracou-Combi station were described in earlier work [18].

### Fungal Material

Strain GY 27 used for inoculation was isolated from an infected pod harvested from clone NA 32, in the Paracou-Combi collection. It was confirmed as belonging to the species *P. palmivora* by studying ITS sequences (using primers ITS 1 and ITS 4). This strain was of sexual compatibility type A2 like all the *P. palmivora* strains isolated in this collection. It displayed the highest level of aggressiveness of all the *P. palmivora* strains isolated in the Sinnamary plots. This primary evaluation was carried out on a range of clones displaying different levels of resistance to this species (data not shown).

The strain was kept in a fungus culture collection by successive transfers every 6 to 8 weeks on diluted V8 medium (200 ml/L). In order to maintain its pathogenicity, strain GY 27 was inoculated approximately every three months on a mature green pod of clone NA 32 and left to incubate at 24°C and at 100% humidity for 4 to 5 days. The strain was then isolated again under sterile conditions on water-agar medium (15 g of agar/L) in a Petri dish, then four days after on 1/5 V8 medium (40 ml/L).

For inoculum preparation (sporocyst and zoospore formation), GY 27 was grown on V8 1/5+ Beta sitosterol medium for 3 days in total darkness at 24°C, then for 7 days in indirect light at 24°C. Zoospores were released after thermal shock (cold water +20 min at 4°C). The zoospore suspension was then calibrated at 300,000 zoospores/ml using a Malassez counting chamber.

### Experimental Protocol

The leaf disc test described by Nyassé [19] and Tahi [11], [16], [17], [20], [21] was used in our study, for its good correlation with losses caused by black pod rot in the field.

In this test, clonal performance in relation to *P. palmivora* was estimated by the appearance and area of the necrotic patches appearing on the leaf discs after inoculation with a calibrated zoospore suspension.

A 10 µl drop of zoospore suspension at a concentration of 300,000/ml was deposited on the underside of each leaf disc. The inoculated discs were placed in trays and incubated in the dark at 25°C. Symptoms were scored after six days’ incubation, using Nyassé’s scale [19]. Resistance levels were defined as follows: Very Resistant (VR: 0< score ≤1), Resistant (R: 1< score ≤2), Moderately Resistant (MR: 2< score ≤2.5), Susceptible (S: 2.5< score ≤3.5), Very Susceptible (VS: 3.5< score ≤5).

Ten series of tests (forming incomplete statistical blocks) were carried out from May 2008 to October 2010, thus covering all seasons and all physiological states of the cocoa trees. Ten incubation trays were used for each series, at a rate of one leaf disc per clone per tray. The leaves used (one or two per clone and per series) were collected at the following stage : mature dark green leaves around 60 days old, with stem just starting turning brown, picked early in the morning, for physiological reasons (non-closure of leaf stomata).

The numbers of clones tested varied from 144 to 190 per series (174.5 on average), depending on the availability of leaves at the stage described above.

### Statistical Methods

We modelled the link between the scores assigned to each disc and the clone using a generalized linear model (GLM) [22] with an ordinal probit link [23]. This model respected the ordinal qualitative nature of the scores, which were equal to 0, 1, 2, 3, 4 or 5 depending on the degree of disc necrosis. The tray effect was integrated into the model to take into account experimental variability (blocks, trays). The significance of the clone and tray effects was assessed by likelihood ratio effect tests (P value  = 0 for each of the effects). As usual, for all tests, an effect is considered significant when P value <0.05.

We carried out likelihood ratio effect tests by pairs to assess clonal differences and construct homogeneity groups. For each pair of clones, we compared the general model, GLM probit, integrating the tray and clone variables, assuming successively: 1) that each clone had a different effect, and 2) that the two clones had an identical effect. All the statistical processing was carried out with R software [24].

## Results and Discussion

The average clonal scores for the 191 objects varied from 1.38 to 3.41, for a general average of 2.35. The “susceptibility indicator” clones were effectively classed as “Susceptible” and even figured among the most susceptible, such as GF 24 (classed next to last with a score of 3.32) and ELP 40-B (2.97), showing that the tests were valid. The two SCA 6 resistance control samples were classed 33rd and 37th, with scores of 1.87 and 1.88, respectively.

Distribution of the raw average scores was as follows: 47 objects (of which 45 Guianan clones) had a score equal to or under 2, 66 objects had a score between 2 and 2.5, and 78 objects had a score over 2.5.

**Figure 1:**
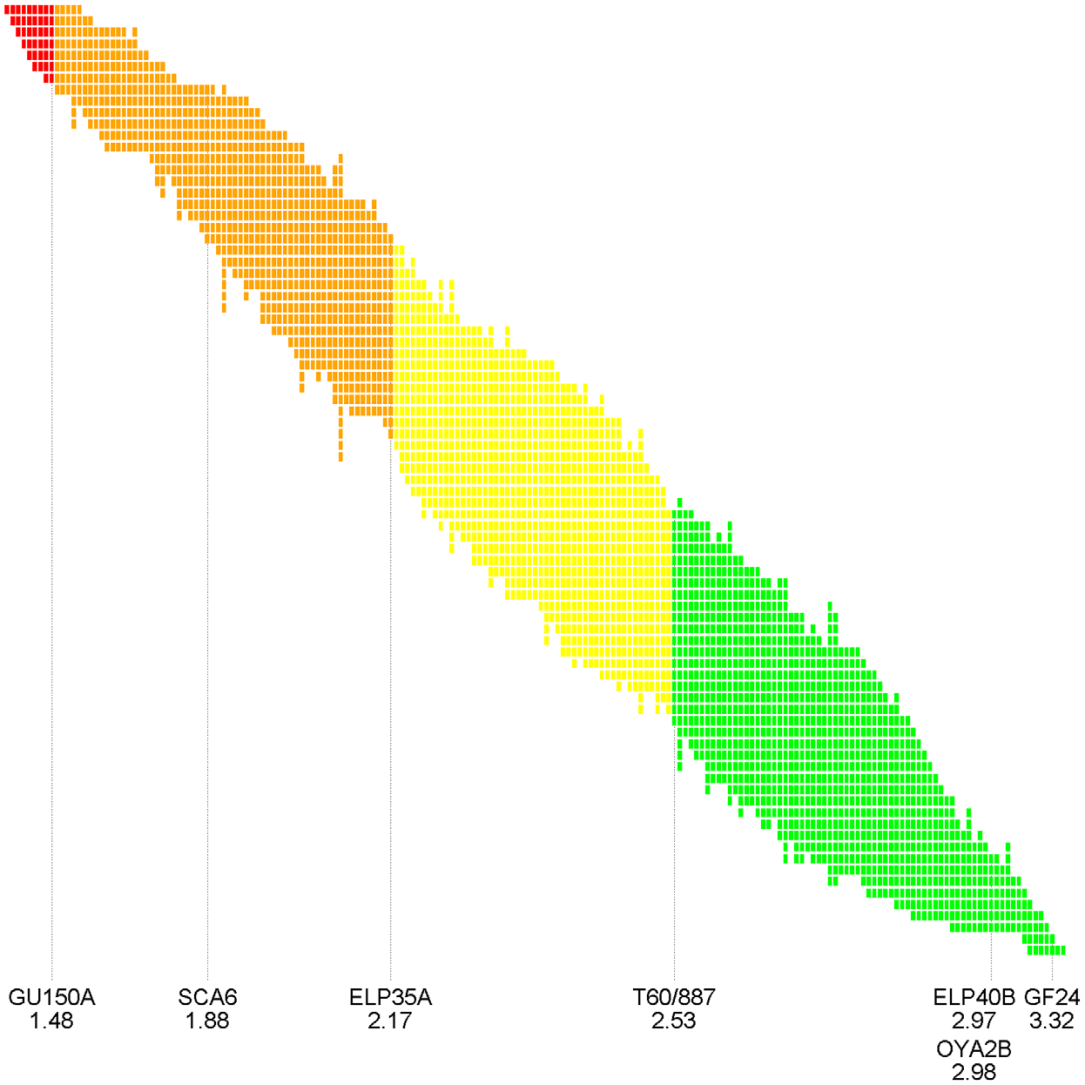
Cross-representation of the clones and homogeneity groups. Each column corresponds to a clone and each row to a homogeneity group. Four statistically different groups are represented, from left to right: very resistant clones  =  more resistant than SCA6 (red); resistant clones  =  equivalent to SCA 6 (orange); moderately resistant clones  =  less resistant than SCA6, but more resistant than ELP 40-B (yellow); susceptible clones  =  not different from ELP 40-B (green). The clones indicated, with their average score, are the controls (SCA 6, ELP 40-B, OYA 2-B, GF 24) and those on the edge of groups (GU 150-A, ELP 35-A, T60/887).

The analysis by the ordinal probit model revealed 83 homogeneity groups (Fig. 1). Sixty-one clones (of which 59 Guianan clones), scored from 1.53 to 2.26, were not statistically different from the SCA 6 control (P value >0.05), whilst 9 clones were statistically more resistant than SCA 6 (Table 2; Fig. 1). Conversely, 71 objects were equivalent to the “susceptibility” indicator clones (of which 68 wild “Guiana” clones) with scores varying from 2.53 to 3.41, and were classed “Susceptible”. Between the two, 50 Guianan clones were therefore “Moderately Resistant” (Table S1). There were no “Very Susceptible” clones. There were therefore 109 “Resistant” and “Moderately Resistant” Guianan clones out of the 186 tested, amounting to a “resistance index” (IRBP, [15]) of 58.6%.

The 24 clones pre-selected for high productivity and low losses caused by black pod rot in the field in French Guiana after 10 years’ monitoring were classed as follows after our 10 series of leaf tests: 11 clones classed as resistant, 6 clones as moderately resistant and 7 clones as susceptible. Two out of the 7 susceptible clones had a score over 3: GU 129-B (3.19) and GU 138-A (3.09).

Seventeen out of 24 clones (71%) were therefore “Resistant” or “Moderately Resistant”, revealing good coherence with the field observations. However, the case of the seven “Susceptible” clones shows that the pod rot diseases in the field were perhaps not all due, at the time, to as virulent a strain as that used in the laboratory, and/or that other factors linked to resistance occurred in the field (short ripening period, inducing an escape phenomenon, for example).

Each of the 10 series (blocks) was very significantly positively correlated (Pearson’s coefficient of correlation) with the general mean, with R values varying from 0.42 to 0.59 (Table S2).

This study enabled us to quantify the level of resistance to *P. palmivora* in 185 Guianan clones belonging to 17 natural populations, along with one subspontaneous clone. At the end of the 10 series of inoculations, the averages of the score for the two samples of the SCA 6 resistance control clone were 1.87 and 1.88, values that were higher than most of those published, showing high aggressiveness for the local strain GY 27. In fact, even though SCA 6 is always resistant, the scores published vary, depending on the conditions, protocols and *P. palmivora* strain used, from 1.19 [12] to 1.92 [8], passing through 1.25 ([13]; local clonal trial in Ghana), 1.30 [11], 1.43 ([13]; clones in the Divo collection, Ivory Coast), 1.56 [14], 1.64 ([13]; families in trials at Divo and Abengourou, Ivory Coast), 1.71 [15] and 1.75 ([13]; international clonal trial in Ivory Coast). It can therefore be deduced that our scores were a little high, which was confirmed by the fact that the group of clones equivalent to SCA 6 included clones whose score (2.26 for the least resistant in the group) exceeded the limit arbitrarily fixed for resistance, i.e. 2 (Table 2).

The distribution of the resistant clones in the demes (Table 2) showed that some demes were not represented, such as Borne 7 (where 4 out of 7 clones were classed “susceptible”), or were under-represented (CAM 9 and KER). Conversely, some demes were more represented in the “Resistants” than in the individuals tested: CAM 1 (26.4% of resistants as opposed to 14.5% of the individuals tested), CAM 7 (14.7 as opposed to 10.2) and CAM 13 (8.8 as opposed to 5.4). The nine clones more resistant than SCA 6 nonetheless belonged to 5 demes: 3 belonged to CAM 1, 2 to CAM 9, 2 to ELP, 1 to CAM 7 and 1 to CAM 13. These observations partly confirmed those reported by Paulin *et al.* [10], as regards resistance to *P. megakarya*, apart from the Borne 7 population.

Our results confirmed those of other works involving the Guianan clones already tested with *P. palmivora*. For instance, GU 255-V, one of the clones in the CFC project international clonal trial [6], [8], received a score of 1.78 in our study, whereas it scored 1.86 in Ivory Coast [13] and 1.88 in Montpellier (CIRAD), where it was classed better than SCA 6 [8]. The same applied for clone GU 175-V, a moderately susceptible clone (2.58 in [8]), which scored 2.68 in our study. Very susceptible clone GU 138-A (scored 3.60 in [9]) was classed 184th out of 191 in our study, with a score of 3.09.

In Ivory Coast, in the CFC-IPGRI project, after 2 series of tests with *P. palmivora*, 15 clones of the “Guiana” group in the Divo collection (out of 16 tested) proved to be resistant, and three were classed ahead of the SCA 6 control [13]. Those three clones (two from deme Cam 7 and one from Cam 1) had five sibs in our study, of which only two were classed “Resistant”, whilst one was “Moderately Resistant” and two were “Susceptible”, seeming to indicate notable within-family variation.

Compared to the results obtained by Paulin *et al.* [10] when studying the resistance of 59 “Guiana” clones to *P. megakarya* using the same methodology, our results (on 53 common clones) showed a positive and significant correlation (Pearson’s coefficient of correlation; R = 0.36; P = 0.009) between the scores obtained, confirming conclusion reached by those authors: overall, a correlation exists for resistance to the two species; nevertheless, the R^2^ value is low.

### Conclusions

The results of our study, using an efficient methodology (ten series of tests, 100 discs sampled per clone over two and a half years, as opposed, in general, to 2 series of 40 discs, an aggressive *P. palmivora* strain, and an appropriate statistical test adapted to the ordinal nature of the data and not a simple ANOVA) confirmed that the “Guiana” genetic group is an important source of resistance to *P. palmivora*. Numerous clones (59) proved to be as resistant as the SCA 6 reference, whilst 9 were statistically more resistant, which is quite rare [14]; indeed, of the internationally used clones, only IMC 47 seems to be more resistant than SCA 6 [8]. Given the strong aggressiveness of strain GY 27, we were able to separate the “Guiana” clones according to their level of resistance with great confidence, and to transpose our results to other producing countries, where only *P. palmivora* is present on cocoa trees, for the introduction of resistant clones.

Some of the “Guiana” clones more resistant than SCA 6 could be incorporated into numerous cocoa breeding programmes, especially those that display other notable qualities too, such as GU 315-A, the best of the GU clones for the mean fresh bean weight per pod and among the best five for average bean weight [5]. The same applies for many other clones equivalent to SCA 6, particularly the “elite” clones GU 134-B, GU 139-A and GU 285-A [5].

As regards Guianan applications, the results presented in this study, along with those to come from tests of resistance to *P. capsici*, witches’ broom (caused by *Moniliophthora perniciosa*) and *Ceratocystis* wilt (caused by *Ceratocystis* spp.), will enable a choice to be made from around ten “elite” clones for use in organic cocoa growing.

All clones tested can be obtained upon request to first or last author, and delivered after a quarantine period and the signature of a Material Transfer Agreement [25]. Some of the resistant clones have already been transferred to a quarantine station and therefore can be available within a shorter period.

## Supporting Information

Table S1 List of moderately and susceptible clones (ordered according to their effect in the ordinal probit model), their average score after 10 series of tests, and their deme.
(XLS)

Table S2 Pearson coefficient of correlation between series of inoculations with *P. palmivora* and between each series and the general mean.
(XLS)
